# A reanalysis of “Two types of asynchronous activity in networks of excitatory and inhibitory spiking neurons”

**DOI:** 10.12688/f1000research.9144.1

**Published:** 2016-08-22

**Authors:** Rainer Engelken, Farzad Farkhooi, David Hansel, Carl van Vreeswijk, Fred Wolf

**Affiliations:** 1 Max Planck Institute for Dynamics and Self-Organization (MPI-DS), Bernstein Center for Computational Neuroscience Göttingen, Faculty of Physics,, University of Göttingen, Göttingen, Germany; 2 Collaborative Research Center 889, University of Göttingen, Göttingen, 37099, Germany; 3 Institut für Mathematik, Technische Universität Berlin and Bernstein Center for Computational Neuroscience, Berlin, Germany; 4 Cerebral Dynamics, Learning and Memory Research Group, Center for Neurophysics, Physiology and Pathology, CNRS UMR8119, Université Paris Descartes, Paris, France

**Keywords:** rate chaos, balanced state, mean field theory, network dynamics

## Abstract

Neuronal activity in the central nervous system varies strongly in time and across neuronal populations. It is a longstanding proposal that such fluctuations generically arise from chaotic network dynamics. Various theoretical studies predict that the rich dynamics of rate models operating in the chaotic regime can subserve circuit computation and learning. Neurons in the brain, however, communicate via spikes and it is a theoretical challenge to obtain similar rate fluctuations in networks of spiking neuron models.

A recent study investigated spiking balanced networks of leaky integrate and fire (LIF) neurons and compared their dynamics to a matched rate network with identical topology, where single unit input-output functions were chosen from isolated LIF neurons receiving Gaussian white noise input. A mathematical analogy between the chaotic instability in networks of rate units and the spiking network dynamics was proposed.

Here we revisit the behavior of the spiking LIF networks and these matched rate networks. We find expected hallmarks of a chaotic instability in the rate network: For supercritical coupling strength near the transition point, the autocorrelation time diverges. For subcritical coupling strengths, we observe critical slowing down in response to small external perturbations. In the spiking network, we found in contrast that the timescale of the autocorrelations is insensitive to the coupling strength and that rate deviations resulting from small input perturbations rapidly decay. The decay speed even accelerates for increasing coupling strength.

In conclusion, our reanalysis demonstrates fundamental differences between the behavior of pulse-coupled spiking LIF networks and rate networks with matched topology and input-output function. In particular there is no indication of a corresponding chaotic instability in the spiking network.

## Introduction

Slow neural dynamics are believed to be important for behavior, learning and memory ( Churchland & Shenoy, 2007; Fee & Goldberg, 2011; Murray *et al.*, 2014). Rate models operating in the chaotic regime show rich dynamics at the scale of hundreds of milliseconds and provide remarkable learning capabilities ( Barak *et al.*, 2013; Sussillo & Abbott, 2009; Toyoizumi & Abbott, 2011). Understanding the conditions of such a transition to chaos in more detailed network models has recently attracted a lot of interest ( Harish & Hansel, 2015; Kadmon & Sompolinsky, 2015). However, neurons in the brain communicate via spikes and it is a challenge in computational neuroscience to obtain similar slow rate dynamics in networks of spiking neuron models.

This question was recently addressed in a paper by Ostojic (2014) published in Nature Neuroscience ( Ostojic, 2014). It argues that an “unstructured, sparsely connected network of model spiking neurons can display two fundamentally different types of asynchronous activity”. When the synaptic strength is increased, networks of leaky integrate-and-fire (LIF) neurons would undergo a *transition* from the “well-studied asynchronous state, in which individual neurons fire irregularly at constant rates” to another “heterogeneous asynchronous state” in which “the firing rates of individual neurons fluctuate strongly in time and across neurons” ( Ostojic, 2014). These two regimes would differ in an essential manner, the rate dynamics being chaotic beyond the phase transition. Finding a transition to chaotic slow-varying rate dynamics in spiking networks in such a simple model would be an important step towards an understanding of the computations underlying behavior and learning and would fill a gap in the current understanding of network dynamics. Here we re-examine the behavior of random LIF networks and demonstrate that there is no such phase transition to chaos in the spiking network analyzed in ( Ostojic, 2014). While we confirm the observed deviation from the mean field theory description that assumes uncorrelated Gaussian fluctuations in time and among neurons, we controvert the validity of the presented analysis. We provide a series of tests of dynamical behavior that refute the existence of a chaotic instability and show that the analogy between the spiking network and the rate network is conceptually misleading and mathematically flawed.

**Figure 1.**
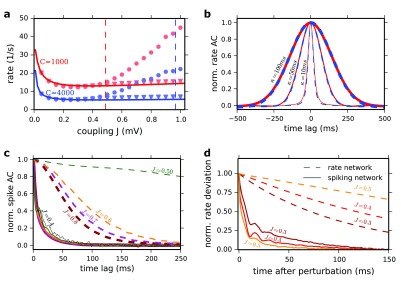
( **a**) Population averaged firing rate in the network vs. coupling strength J. Solid lines: Ricciardi mean field for C=1000 (red) and C=4000 (blue). Predictions for J _c_ (Equation 16) are indicated by the corresponding dashed vertical lines. Simulation results (event-based simulation implemented in Julia programing language) are also plotted. Dots: Δ=0.55 ms synaptic delay. Triangles: Δ=0.0 ms. Results for C=1000, N=10000 (red marker) and C=4000 and N=40000 (blue marker). ( **b**) Averaged normalized AC of neuronal rate functions for J=0.8 mV and C=1000 (red) and C=4000 (dashed blue) LIF networks. The rate functions were computed by filtering the spike trains of the neurons (1 ms time bin) with a Gaussian filter with 10 ms (the thinnest lines), 50 ms (moderated lines) and 100 ms (the thickest lines) standard deviation. ( **c**) Autocorrelation function of the spike trains (no filtering) normalized to the second pick. Solid lines: LIF network. Dashed lines: Poisson network. The results are shown for J = 0.5 mV (dark green), J=0.6 mV (dark orange), J = 0.7 mV (magenta) and J=0.8 mV (dark red). For the LIF the AC is also shown for J=0.4 mV (solid black). To compute the ACs for the Poisson network we simulated a network for 100 s (time step 1 ms) and averaged the results over 40 realizations of the initial conditions. The network size is N=100000 for 0.5≤J≤0.6mV and N=10000 for J>0.6 mV. For the LIF network we averaged spike autocorrelation of 3000 randomly chosen neurons with a 1 ms bin following Equation 23 in the paper. All parameters are as in Figure P3. ( **d**) Subcritical behavior of the systems. Rate network and spiking network are both perturbed in the constant feed-forward input current *µ*_0_ in the least stable direction of the linearized rate dynamics (Equation 16) for different coupling strengths J. The resulting rate deviation is projected onto the perturbation direction. Dashed lines reflect the normalized decay of this perturbation in the rate network and the solid lines those of the spiking network (averaged over 1.42 million perturbations). The perturbation was applied to the constant feed-forward input *µ*_0_ for 2 ms where the standard deviation of the perturbation vector was 1 mV. Longer perturbation durations (10 ms) and weaker perturbation strengths (standard deviation 0.1 mV) gave very similar results (not shown). Perturbation direction, strength, duration and network realization were exactly the same for rate and spiking network. Other parameters as in ( Ostojic, 2014).

The paper ( Ostojic, 2014) starts with simulations of a network of LIF neurons for different values of the synaptic strength, J, while all other parameters are fixed to specific values. It is observed that the population mean firing rate of the neurons, *ν*_0_, is well described by a mean field calculation only below a certain coupling strength J*. At this value, the average firing rate starts to deviate from the mean field prediction more than 5%. (Figure 1a in ( Ostojic, 2014), denoted Figure P1a; hereafter figures in ( Ostojic, 2014) are denoted by their numbers preceded by a “P”). In ( Ostojic, 2014), it was claimed that the “classical” asynchronous state exhibits an instability at J=J*. Above J* the dynamics would still be asynchronous, but in a way which would be essentially different from the “classical” asynchronous state. To assess this claim, the author replaced the full dynamics of the spiking LIF network by a rate model of similar connectivity, the “Poisson network”. Simulations indicate that as J increases, there is a value, J=J _c_, at which the dynamics of the latter undergo a phase transition between a state in which the rates are constant in time (fixed point) and a state in which they fluctuate chaotically with long network generated time-scales. The author then derives an equation for a critical value J _c_ which is in agreement with the simulations of the Poisson model. For the parameters used in Figure P1 and P2 the value of J _c_ is rather close to J*. Apparently the author felt that this similarity, gives sufficient reason to justify two conclusions: (i) in the LIF network an instability occurs near J* which is of the same nature as the one occurring at J _c_ in the Poisson network. (ii) The asynchronous states below and above J* are essentially different in the LIF network.

However, as we now show, the reported agreement between the predicted transition at J _c_ and the spiking network simulation results is coincidental and only valid for the chosen parameters used in the paper ( Ostojic, 2014) but not in general. We start by providing two counter-examples to statements (i) and (ii).

## Methods and results

Our first counter-example is the LIF model considered in ( Ostojic, 2014), we take N=40000 neurons and C=4000 synapses per neuron instead of N=10000 and C=1000 (all other parameters as in Figure P1, except for the network size, keeping the connection probability constant). The population firing rate, *ν*_0_ (J), is plotted in Figure 1a. It deviates from the mean field prediction at J*≅0.3 mV by more than 5%. Nonetheless, the critical point in the corresponding Poisson rate network is J _c_≅0.96 mV and thus it is more than three times larger than J*.

**Figure 2.**
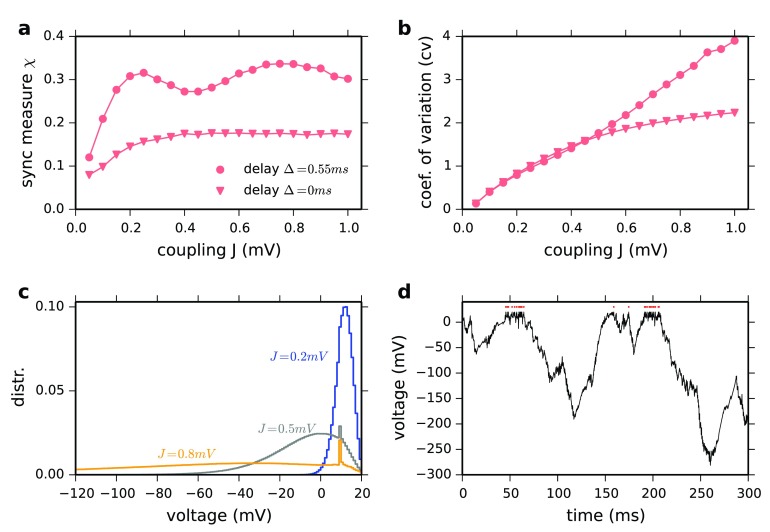
( **a**) Synchrony measure X vs. coupling strength J. Dots: Δ=0.55 ms. Triangles: Δ=0.0 ms for N=10000, C=1000. X is defined as in ( Hansel & Mato, 2003) on the phases of neurons. Note that zero delay does not increase network synchrony. ( **b**) Coefficient of variation of the interspike intervals vs. coupling strength J. ( **c**) Distribution of membrane potentials for different coupling strength J (in mV). ( **d**) Example voltage trace for J=0.8 mV shows very negative excursions followed by short bursts of action potentials. Red dots indicate spike times. Numerically exact event-based simulation were implemented in Julia programing language. Other parameters are chosen as in ( Ostojic, 2014).

Our second counter-example is the LIF network of Figure P1 and P2 with the same parameters except for the delay, Δ. We note that the delay does not affect the existence of the asynchronous state and importantly plays no role in the mathematical considerations of Ostojic (2014). As these yield identical results irrespective of delay we consider the simplest case: Δ = 0 ms. Strikingly, the spiking network shows no longer a large deviation from the mean-field prediction ( Figure 1a). However, the proposed analogy with the Poisson rate network still predicts that a deviation should occur at J*≅0.49 mV, since the transition to chaos in the Poisson network is independent of the delay. The author seems to be somewhat aware of this discrepancy. Indeed, it is stated in the Online Methods that delays must be larger than the refractory period, because “if the delays are shorter, spikes that reach a neuron while it is refractory do not have an effect and the overall coupling is effectively reduced” ( Ostojic, 2014). If this was correct, this effective reduction should be reflected in the formula for predicting J _c_ (Equation 16). This is not the case: the latter does not depend on Δ. In addition, the spiking network for Δ = 0 ms in fact exhibits no increased level of network synchrony measured by the common synchrony measure X ( Figure 2a) ( Hansel & Mato, 2003).

It is also argued in the paper ( Ostojic, 2014) that the results plotted in Figure P3a and b support the analogy between the rate dynamics of the Poisson model and the dynamics of the LIF network. However, the comparison made in this figure is conceptually misleading. In the Poisson model, the rate as a function of time is an *unequivocally* defined quantity. It is *the dynamical variable* of the model and the time scale over which the rate fluctuates for strong enough coupling is fully determined by these dynamics. This is not the case in the LIF model where the “rate” and its “dynamics” depend on the temporal width over which the spiking activity is filtered. The width of the Gaussian filter used in ( Ostojic, 2014) is 50 ms. This choice is arbitrary and is the reason for the similarity observed in the rate autocorrelations (ACs) plotted in the upper and lower panels in Figure P3b which depends on this choice ( Figure 1b). The rate functions were computed by filtering the spike trains of the neurons (1 ms time bin) with a Gaussian filter with 10 ms (the thinnest lines), 50 ms (moderated lines) and 100 ms (the thickest lines) standard deviation. Moreover, the spike ACs plotted in Figure P3c for the two models exhibit essential differences as we now show.

**Figure S1.**
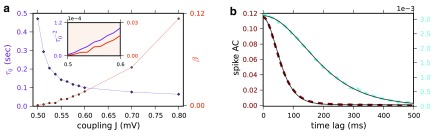
( **a**) The decorrelation time ( *τ*_0_, violet diamond, left y-axis) and amplitude at zero time lag (beta, orange circles, right y-axis) of the baseline-subtracted population averaged spike AC are plotted vs. J for the Poisson network. These parameters were obtained by fitting the spike AC with *ACF*( *τ*) = *β/cosh*( *τ/τ*_0_) ^2^ (see Figure S1b). Inset: the rescaled estimated *τ*_0_^-2^ (left axis, violet) and β values (orange, right axis) for J=0.5, 0.5125, 0.525, 0.5375, 0.55, 0.5625, 0.575, 0.5875 and 0.6 mV, to show that they vanish linearly near the phase transition. ( **b**) The non-normalized spike AC can be very well fitted by *ACF*( *τ*) = *β/cosh*( *τ/τ*_0_) ^2^. Dashed lines: Simulation results; J=0.525 mV (cyan, right y-axis) and J=0.8 mV (dark red, left y-axis): Black solid line: The fits.

For J=0.2 and 0.4 mV, the spike AC in the Poisson rate model (Figure P3c, upper panel) is close to a Dirac function reflecting that the dynamics are at fixed point - that is the rate variable from which the Poisson process of the spikes is generated is constant. For J=0.6 mV the spike AC is very different: a broad component has now appeared. It is flat at zero time lag and has a negative curvature at short time lags ( Figure 1c and Figure P3c). A detailed analysis reveals that this change has all the characteristics of a true phase transition. It shows that close to the phase transition, the amplitude vanishes proportionally to J-J _c_ and the decorrelation time diverges as $1/\sqrt{J–J_{c}}$ ( Figure S1a). To compute the ACs for the Poisson network we simulated a network for 100 s (time step 1 ms) and averaged the results over 40 realizations of the initial conditions. The network size is N=100000 for 0.5≤J≤0.6mV and N=10000 for J>0.6 mV. For the LIF network we averaged spike autocorrelation of 3000 randomly chosen neurons with a 1 ms bin following Equation 23 in the paper. All parameters are as in Figure P3.

The spike AC behaves very differently in the LIF network. For J=0.2 mV it exhibits at zero time lag a sharp peak flanked by a trough which reflects the refractoriness (absolute and relative) of the single neuron dynamics. As J increases, there is a progressive change in the AC shape. Eventually, the trough disappears. The flanks of the zero peak are now decreasing exponentially ( Figure 1c, solid lines). A careful analysis reveals that the typical time constant of this decrease depends only weakly on J ( Figure 1c, solid lines). It is always on the order of the membrane time constant of the neurons (20 ms). Note also that by contrast with what is observed in the Poisson network, for J=0.5 to 0.8 mV, the spike AC curvature is always positive and peaked around zero time lag ( Figure 1c, dashed lines).

How do the “strong fluctuations” in the “heterogeneous regime” emerge? For increasing J, the spiking activity of single neurons becomes increasingly irregular, quantified by the mean coefficient of variation (cv) of the interspike interval distribution ( Figure 2b). At the same time, the distribution of membrane potentials develops a very long tail towards negative voltages ( Figure 2c). For strong coupling (J=0.8 mV), voltage traces of individual neurons show long very negative voltage excursions, followed by short bursts of action potentials ( Figure 2d). This explains the super-Poissonian irregularity (CV>1). The super-Poissonian nature of spiking irregularity and the unphysiological negative voltage deviations are properties related to the linear V̇-V-relationship of the LIF model. A mean-field description of this phenomenon requires self-consistent spike train autocorrelations ( Lerchner *et al.*, 2006; Wieland *et al.*, 2015). For other integrate-and-fire neurons e.g. the quadratic-integrate-and-fire model, even for very strong coupling J, e.g. J = 20 mV, the mean coefficient of variation does not increase beyond one and no strongly negative voltage excursions are observed. All parameters are as in Figure P1.

Additionally, in order to compare the behavior of spiking and rate models below the postulated phase transition, we perturbed rate and spiking networks of identical topology in the least stable direction of the linearized rate dynamics, predicted by Equation 16 in the paper ( Ostojic, 2014). The resulting rate deviation is projected onto the perturbation direction. The perturbation was applied to the constant feed-forward input *µ*_0_ for 2 ms where the standard deviation of the perturbation vector was 1 mV. Figure 1d shows that the decay of the perturbation in the rate network slows down near the transition, indicating a critical slowing down ( Figure 1d, dashed lines). If there were a “mathematically analogous” transition in the spiking network, also its perturbation should decay slower as the transition is approached. Our result ( Figure 1d, solid lines) shows that the decay time-scales of the perturbation (averaged over 1.42 million perturbations) is insensitive to J and it stays close to the membrane time constant (similar to solid lines in Figure 1c). Longer perturbation durations (10 ms) and weaker perturbation strengths (standard deviation 0.1 mV) gave very similar results (not shown). All other parameters are chosen as in ( Ostojic, 2014).

## Conclusion

We therefore conclude that, contrary to what was argued by the author, the spiking LIF network studied in ( Ostojic, 2014) does not exhibit a phase transition to a chaotic state similar to the one occurring in the studied rate model. The reported mismatch between the average firing rate in this LIF network simulations and the mean-field calculation is unrelated to such a transition.

## Data and software availability

*Zenodo*: Reanalysis of “Two types of asynchronous activity in networks of excitatory and inhibitory spiking neurons”, 10.5281/zenodo.59624 ( Engelken & Farkhooi, 2016).

The data for this article are also available on the Open Science Framework at: https://osf.io/q3vt4/
