# Supplementary material for: A reanalysis of “Two types of asynchronous activity in networks of excitatory and inhibitory spiking neurons”
Source: F1000Res. 2016 Aug 22;5:2043. [Version 1] doi: 10.12688/f1000research.9144.1 (PMC5040152; doi:10.12688/f1000research.9144.1)
Supplement: Peer review history

# Peer review: Referee response for version 1

**DOI:** 10.5256/f1000research.9839.r16565

**Published:** 2016-09-27

**Authors:** Maoz Shamir

**Affiliations:** 1 Department of Physiology and Cell Biology, Ben-Gurion University of the Negev., Be'er-Sheva, Israel

The origin and possible computational role of neuronal noise has been the focus of considerable scientific effort during the past decades. In particular, the transition to chaos has been extensively studied using simplified rate-models and much is known about this transition.

 A recent work studied the dynamics of a sparsely connected network of excitatory and inhibitory spiking neurons in the balance regime and a compelling mapping between spiking neural network and rate model was proposed. Following analysis and numerical simulations it was suggested that a novel type of asynchronous state exists and it was further hypothesized that this novel state is useful for complex information processing in the central nervous system.

 Here the authors reevaluate this claim. Several counter examples are provided to prove that the claim of a transition to a second type of asynchronous state does not hold. Furthermore, the origin of ‘strong fluctuation’ super-Poisson irregular firing is studied and is found to be related to extremely negative voltage fluctuations that are beyond the typical physiological range.

 The work is timely. The results are solid and well presented. I also find the effort devoted to reproduce and re-evaluate results refreshing. I believe this paper will contribute to the scientific debate.

I have read this submission. I believe that I have an appropriate level of expertise to confirm that it is of an acceptable scientific standard.

# Peer review: Referee response for version 1

**DOI:** 10.5256/f1000research.9839.r15814

**Published:** 2016-09-07

**Authors:** Jonathan Touboul

**Affiliations:** 1 Mathematical Neuroscience Team, CIRB - Collège de France, Oaris, France

Characterizing the dynamics of spiking neural networks and their transitions is currently a prominent issue in computational neuroscience. The question is largely non-trivial and riddled with subtleties: numerical simulations are often delicate to interpret, and theoretical tools to analyze these dynamics are still being developed. 

 The theoretical community have devoted important effort to address this question. Indeed, progresses on this question would advance our understanding of the brain and its computations. A question of particular interest is to characterize phase transitions to chaotic regimes in spiking networks. Indeed, since the seminal work of Sompolinsky, Crisanti and Sommers ^1^ on rate networks, chaotic regime were shown to have rich dynamics able to support efficient computations and learning (see e.g. Sussillo & Abbott 2009 ^2^). Whether spiking networks do show a similar transition and thus share similar properties as rate networks has recently been the focus of several researches and is an important endeavor in computational neuroscience ^3^.

 The present paper addresses a few important questions on the interpretations and conceptual approach of a theoretical article appeared in Nature Neuroscience in 2014 ^4^ dealing precisely with dynamics and transitions in spiking networks. That paper argued for the existence a transition in a balanced spiking network, between an asynchronous and a "new highly fluctuating regime", using in particular transitions of an associated rate model. The present article comes back to this comparison between spiking and rate network, and argues that, in contrast with the approach of Ostojic (2014) ^4^, it is not possible to extract accurate information on the spiking network from an analysis of the particular rate network studied, by showing a mismatch between their qualitative dynamics. Moreover, using relevant numerical quantities to identify phase transitions (synchrony measure and effect of perturbations), the authors establish the absence of phase transition in the spiking network, while the rate network does present the hallmarks of phase transitions. 

 The present paper thus contributes to an important scientific debate on the characterization of the dynamical regimes of spiking networks. This question has been the topic of very recent important works that advance our understanding of spiking networks and the associated mean-field limits (to cite a few, see Kadmon & Sompolinsky 2015 ^5^, Harrish & Hansel 2015 ^6^,  Goedeke, Schuecker & Helias 2016 ^7^). 

 For its contribution to the scientific debate on a timely and important topic in theoretical neuroscience, this paper shall be helpful to the readers interested in the existence and nature of transitions in spiking networks.

I have read this submission. I believe that I have an appropriate level of expertise to confirm that it is of an acceptable scientific standard.

## References

[1] SompolinskyHCrisantiASommersHJ: Chaos in random neural networks. *Phys Rev Lett*.1988;61(3) : 10.1103/PhysRevLett.61.259 259-262 10.1103/PhysRevLett.61.259 10039285

[2] SussilloDAbbottLF: Generating coherent patterns of activity from chaotic neural networks. *Neuron*.2009;63(4) : 10.1016/j.neuron.2009.07.018 544-57 10.1016/j.neuron.2009.07.018 19709635PMC2756108

[3] AbbottLFDePasqualeBMemmesheimerRM: Building functional networks of spiking model neurons. *Nat Neurosci*.2016;19(3) : 10.1038/nn.4241 350-5 10.1038/nn.4241 26906501PMC4928643

[4] OstojicS: Two types of asynchronous activity in networks of excitatory and inhibitory spiking neurons. *Nat Neurosci*.2014;17(4) : 10.1038/nn.3658 594-600 10.1038/nn.3658 24561997

[5] KadmonJSompolinskyH: Transition to Chaos in Random Neuronal Networks. *Physical Review X*.2015;5(4) : 10.1103/PhysRevX.5.041030 10.1103/PhysRevX.5.041030

[6] HarishOHanselD: Asynchronous Rate Chaos in Spiking Neuronal Circuits. *PLoS Comput Biol*.2015;11(7) : 10.1371/journal.pcbi.1004266 e1004266 10.1371/journal.pcbi.1004266 26230679PMC4521798

[7] GoedekeSSchueckerJHeliasM: Noise dynamically suppresses chaos in random neural networks. *arXiv:1603.01880 [q-bio.NC]*.2016; Reference source
